# Genomic analysis of Klebsiella aerogenes circulating in New Mexico

**DOI:** 10.1099/mgen.0.001650

**Published:** 2026-02-24

**Authors:** Leslie M. Huggins, Rachel Sidebottom, William Johnson, Kurt Schwalm, Karissa Culbreath, Jesse Young, Meghan Brett, Darrell L. Dinwiddie, Daryl Domman

**Affiliations:** 1 Clinical and Translational Science Center, University of New Mexico Health Sciences Center, Albuquerque, USA; 2 Department of Pediatrics, University of New Mexico Health Sciences Center, Albuquerque, USA; 3 TriCore Reference Laboratories, Albuquerque, USA; 4 Department of Internal Medicine, University of New Mexico Health Sciences Center, Albuquerque, USA; 5 College of Population Health, University of New Mexico Health Sciences Center, Albuquerque, USA; 6 Bioscience Division, Los Alamos National Laboratory, Los Alamos, USA

**Keywords:** epidemiology, genomics, *Klebsiella aerogenes*

## Abstract

*Klebsiella aerogenes* is an opportunistic pathogen and a growing cause of healthcare-associated infections, characterized by multidrug resistance and the emergence of global high-risk clones. However, regional genomic surveillance data remain limited. Here, we sought to characterize the population structure, transmission dynamics and resistance mechanisms of clinical *K. aerogenes* in Albuquerque, New Mexico. We sequenced 177 clinical isolates collected between 2021 and 2023. We also developed a novel, species-specific PopPUNK database to facilitate rapid, high-resolution typing. The New Mexico *K. aerogenes* population was diverse but dominated by two global pandemic lineages, ST93 (47.5%) and ST4 (7.9%), which were significantly enriched for the virulence factors yersiniabactin and colibactin. Genomic evidence for recent local transmission was rare, with only four putative transmission pairs identified. The resistome was characterized by intrinsic and adaptive mutations. Nearly all isolates possessed *gyrA* mutations associated with decreased fluoroquinolone susceptibility. Mutations in the AmpC regulator AmpD and the outer membrane porin Omp36 were common, particularly within the dominant ST93 lineage. These mutations have been associated with increased AmpC-mediated carbapenem resistance. Our findings underscore the critical importance of genomic surveillance to monitor the transmission and evolution of adaptive resistance.

**Impact Statement**

*Klebsiella aerogenes* is a multidrug-resistant opportunistic pathogen and an escalating public health threat. This study provides the first comprehensive genomic surveillance of *K. aerogenes* in the southwestern USA, revealing that New Mexico’s population is dominated by two globally distributed pandemic lineages (ST93 and ST4). Importantly, we document adaptive chromosomal mutations in regulatory genes (*ampD*) and outer membrane porins (*omp36*) associated with carbapenem resistance. Furthermore, we found that isolates from serially sampled individuals exhibit unexpected genetic diversity, suggesting that initial infections may be polyclonal. To facilitate future surveillance efforts, we developed a novel, species-specific PopPUNK database that provides flexible, rapid lineage assignment. This work establishes a genomic baseline for surveillance in an underrepresented geographic region and demonstrates the critical importance of whole-genome sequencing in tracking adaptive resistance evolution.

## Data Availability

Sequencing data from this project have been deposited under NCBI BioProject PRJNA1338239 and individual accessions found within Table S2. The PopPunk database is available at: https://github.com/DommanLab/Kleb_aero_NM.

## Introduction

*Klebsiella aerogenes* is an increasingly important opportunistic, multidrug-resistant Gram-negative pathogen that is commonly reported as a hospital-acquired infection associated with a wide range of clinical infections, including bloodstream infections, pneumonia and urinary tract infections (UTIs) [1,2]. This bacterium is in the order *Enterobacterales* and was previously known as *Enterobacter aerogenes* until 2017, when whole-genome data were used to determine it was more closely related to species within the genus *Klebsiella* [3]. Although it has been reclassified, the terminology *E. aerogenes* is still frequently used within clinical settings [2]. Infections caused by *K. aerogenes* are linked to particularly poor clinical outcomes. Prior studies have shown that patients with *K. aerogenes* bloodstream infections are significantly more likely to suffer from complications like septic shock, acute kidney injury or death compared to those with similar infections caused by the closely related *Enterobacter cloacae* complex [4].

A defining clinical feature of *K. aerogenes* is the presence of an intrinsic, chromosomally encoded AmpC beta-lactamase [5]. The *ampC* gene is inducible, and its expression can be significantly increased in the presence of certain beta-lactam antibiotics, such as third-generation cephalosporins [5]. This induction can lead to the emergence of resistance during treatment, making the choice of antibiotics critical. For this reason, cefepime, which is a weak inducer of AmpC and is stable against its hydrolytic activity, is often considered a more reliable treatment option than third-generation cephalosporins for serious infections [6]. Carbapenem-resistant *K. aerogenes* (CR-KA) poses a serious clinical challenge and public health threat [7,8]. Carbapenem resistance in *K. aerogenes* occurs through two distinct mechanisms. The first involves acquisition of plasmid-borne carbapenemase genes, including blaKPC, blaNDM and blaOXA-48 [8]. The second and more prevalent mechanism is carbapenemase-independent resistance, resulting from adaptive chromosomal mutations [7]. This pathway involves hyperproduction of the AmpC beta-lactamase due to mutations in regulatory genes like *ampD*, combined with reduced membrane permeability from mutations affecting outer membrane porins such as Omp35 and Omp36 [7,9].

Here, we investigated the genomic diversity, transmission dynamics and antimicrobial resistance of *K. aerogenes* in New Mexico following reports from clinical collaborators of increasing case numbers across multiple hospitals in the Albuquerque metropolitan area (population ~1 million). Further, data reported by the New Mexico Department of Health indicate that the number of reportable carbapenem-resistant (CR) *Enterobacterales* has more than doubled since they began surveillance in 2015 [10], with *K. aerogenes* infections in the top five reported CR organisms in the state. Concerningly, only 6 of the total 52 reported CR-KA had a detectable carbapenemase gene, indicating that 93% of these isolates exhibited carbapenemase-independent resistance [10]. We present the first genomic epidemiological investigation of *K. aerogenes* in New Mexico.

## Methods

### Sample collection and sequencing

We obtained 184 clinical isolates of *K. aerogenes* from TriCore Reference Laboratory, representing two major hospitals and multiple non-hospital medical facilities across Albuquerque, New Mexico. Isolates were collected between September 2021 and May 2023. De-identified clinical data for the isolates were also provided and included the age and sex of the patient, the medical facility (hospitals=A or B and non-hospital clinics=C), date of collection and site of collection (e.g. urine, sputum, blood, etc.). DNA was extracted from each isolate using either the Fisher Scientific KingFisher automated DNA extraction platform or the Zymo DNA mini-prep kits. Sequencing libraries were prepared using Illumina DNA-prep kits and sequenced on the Illumina NextSeq 2000 platform. This study was reviewed and performed according to the University of New Mexico Health Sciences IRB 20-096.

### Genomic and phylogenetic analyses

Illumina short-read data were QCed using *fastp* v0.23.4 [11] and evaluated via *fastqc* v0.12.1 (https://www.bioinformatics.babraham.ac.uk/projects/fastqc/), assembled via *shovill* v1.1.0 (https://github.com/tseemann/shovill) using the SPAdes assembler v3.15.5 [12] and annotated via *prokka* v1.14.6 (https://github.com/tseemann/prokka) all within the *Bactopia* v3.1.1 pipeline [13] using the default parameters. Multilocus sequence typing (MLST) was performed using *mlst* v. v2.23.0 (https://github.com/tseemann/mlst). Quality control and assembly were performed using Bactopia’s default parameters. Samples were failed by Bactopia QC if the total base count was <2.24 Mbp (representing 20× coverage of the smallest bacterial genome size of 112,091 bases), or if assemblies had a total genome size smaller than 100 kbp. After initial processing, genomes were further assessed for quality using CheckM v.1.2.2 [14] using the percent of completion (>95%), contamination (<5%) and the N50 (>100 Kb). This provided us with 177 high-quality genomes for this study. We used *AMRFinderPlus* v3.12.8 [15] to identify antimicrobial resistance and virulence genes. Additional *Klebsiella*-specific virulence and AMR genes were identified via *Kleborate* v3.1.3 [16].

We used *snippy* v.4.6.0 (https://github.com/tseemann/snippy) to map, call variants and generate consensus genome alignments against the type strain *K. aerogenes* KCTC_2190 (NCBI accession NC_015663). *Gubbins* v.3.4 [17] was used to remove putative recombination sites from the consensus alignment. The final consensus SNP alignment was 430,541 sites for the 177 NM isolates. Pangenome and core genome analysis was conducted using Panaroo v.1.5.1 [18]. Maximum likelihood trees were calculated under the GTR+GAMMA model in IQ-Tree v.1.6.12 [19] with 10,000 ultrafast bootstraps and 10,000 bootstraps for the SH-like approximate likelihood ratio. Initial phylogenetic trees and metadata were visualized with MicroReact [20]. Final visualizations were created with Python using the *baltic v.0.3.0* package (https://github.com/evogytis/baltic). GrapeTree v.1.5.037 [21] was used to create minimum-spanning trees, where we collapsed nodes at 30 SNPs. Contextual genomes were pulled from the National Center for Biotechnology Information (NCBI) Pathogens site as assemblies and processed with *Bactopia* as above.

### Flexible PopPunk lineage assignment for *K. aerogenes*

To create a PopPUNK [22] database for *K. aerogenes*, we began with 933 Illumina samples from Feng *et al.* [23] (Table S1, available in the online Supplementary Material) and processed them uniformly using the *Bactopia* pipeline [13], using the same pipeline parameters that were used for the New Mexico isolates. We sketched all genomes using *K*-mer sizes: 13, 17, 21, 25 and 29. After running these sequences through the PopPUNK quality control and database pipelines, our final database included 762 high-quality, informative genomes, all of which had corresponding core-genome MLST (cgMLST) classifications (Table S1). For model fitting, we used the Density-Based Spatial Clustering of Applications with Noise (DBSCAN) algorithm (poppunk --fit-model dbscan) to identify strain clusters based on the distribution of core and accessory genome distances. We provide this database for others to use via our GitHub (https://github.com/DommanLab/Kleb_aero_NM).

## Results

### *K. aerogenes* cases in New Mexico

**Fig. 1.**
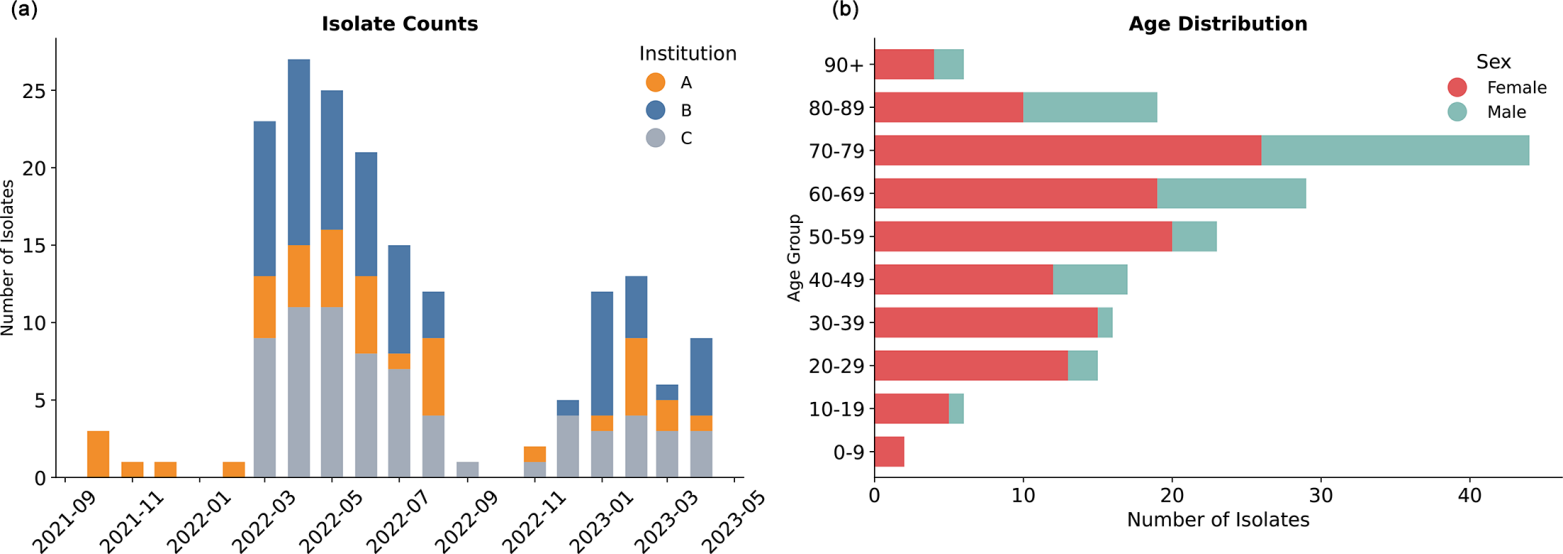
Temporal and demographic characteristics of the *K. aerogenes* isolates sampled in New Mexico. (a) Month of collection for isolates and healthcare institutions. Cases span from 2021 to 2023 and are coloured according to the institution that was the origin of the sample. Institutions A, B and C represent anonymized healthcare facilities. (b) Age and sex distribution of cases. Horizontal stacked bar chart showing the number of cases by 10-year age groups, stratified by sex.

We received 177 *K. aerogenes* isolates from clinical cases in Albuquerque, New Mexico, between October 2021 and April 2023 from two large hospitals (de-identified as Institutes A and B) and non-hospital medical facilities (Institute C). Patient ages ranged from 0 to 100 years, with 47.5% (*n*=84) aged over 65 (Fig. 1). The majority of cases were female (*n*=126, 71.2%). Most isolates were obtained from urine cultures (83.1%, *n*=147), with smaller numbers from blood (*n*=9), wounds (*n*=5) and fluid (*n*=5), among other sources (Fig. S1). Cases were relatively evenly distributed across the three sampling sites: 39% from non-hospital facilities (*n*=69), 38% from Hospital B (*n*=68) and 23% from Hospital A (*n*=40). Within this dataset, 15 individuals provided multiple isolates: 13 patients provided 2 isolates each, and 2 patients provided 3. Of note, the temporal distribution of isolates (Fig. 1a) represents the samples available for sequencing rather than a comprehensive measure of *K. aerogenes* incidence. The lower number of isolates observed in 2021 likely reflects altered healthcare utilization patterns during the SARS-CoV-2 pandemic, followed by a return to typical submission levels in 2022.

### Flexible PopPunk lineage assignment for *K. aerogenes*

A PopPUNK database leverages whole‐genome *k*-mer distances to capture both core and accessory genomic variation [22]. This method provides a faster, more flexible and computationally efficient means of clustering and assigning new isolates compared to the gene-focused cgMLST scheme, and it often better reflects genome-wide evolutionary and epidemiological patterns while complementing the high resolution of predefined core loci in cgMLST. Using the DBSCAN clustering model (see Methods), our final PopPUNK database comprised 762 high-quality *K. aerogenes* genomes organized into 286 unique PopPUNK (PP) clusters. These clusters corresponded to 283 described sequence types (STs) and 180 cgMLST clonal complexes (Fig. S2) described by Feng *et al.* [23]. Overall, 89.9% of all samples (685/762 isolates) were assigned to PP clusters containing a single cgMLST group, and 99.0% of PP clusters (283/286) showed perfect one-to-one correspondence with a single cgMLST clonal complex.

The 286 PP clusters exhibited considerable variation in size, with 170 singleton clusters (59.4%) representing unique or poorly sampled lineages, 91 small clusters (2–5 isolates), 22 medium clusters (6–20 isolates) and only 3 large clusters containing more than 20 isolates. The two largest clusters were PP-1_125 (54 isolates, ST93/CC3) and PP-2 (52 isolates, ST4/CC2), which together accounted for 13.9% of all genomes in the database, followed by PP-3 (27 isolates, ST135/CC4). While the vast majority of PP clusters had one-to-one correspondence with cgMLST clonal complexes, three PP clusters each merged multiple cgMLST groups, affecting 77 isolates (10.1% of the dataset): PP-1_125 unified CC1 and CC3 (54 isolates total, with CC1 representing 63.0% and CC3 representing 37.0%); PP-9 merged CC55, CC76 and CC191 (10 isolates); and PP-6 combined CC13 and CC35 (13 isolates). Conversely, 45 cgMLST clonal complexes (25.0%) were split across multiple PP clusters, with the most fragmented being CC8 (21 isolates across 14 PP clusters), CC5 (20 isolates across 10 PP clusters) and CC7 (21 isolates across 9 PP clusters).

### Diversity of New Mexico isolates

**Fig. 2.**
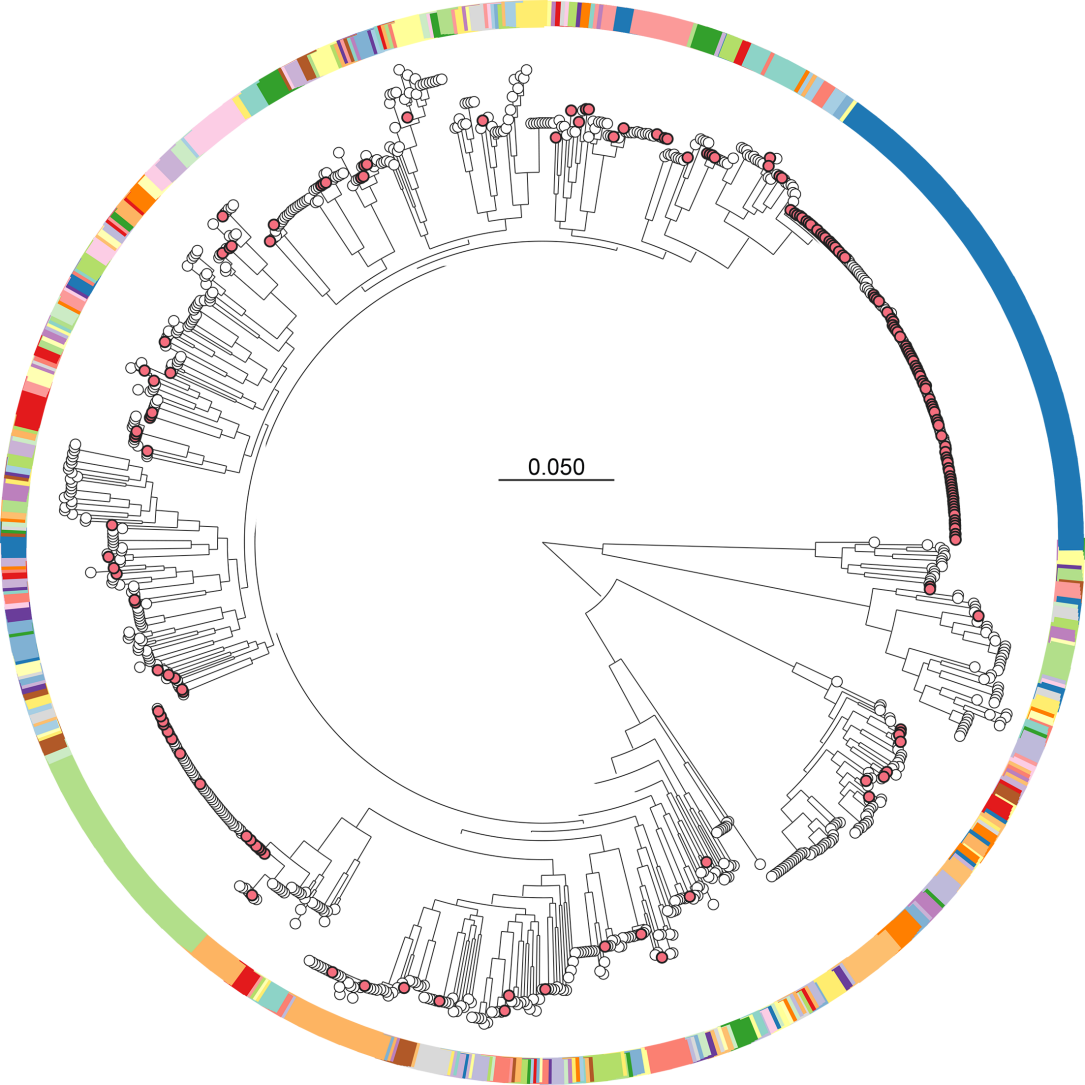
Maximum likelihood phylogenetic tree showing the diversity of *K. aerogenes* isolates sampled from New Mexico. The New Mexico isolates (*n*=177) are shown as red nodes, set within the context of global contextual strains (*n*=762). The colour bands correspond to PopPunk clusters. Scale bar denotes number of substitutions per variable site.

**Fig. 3.**
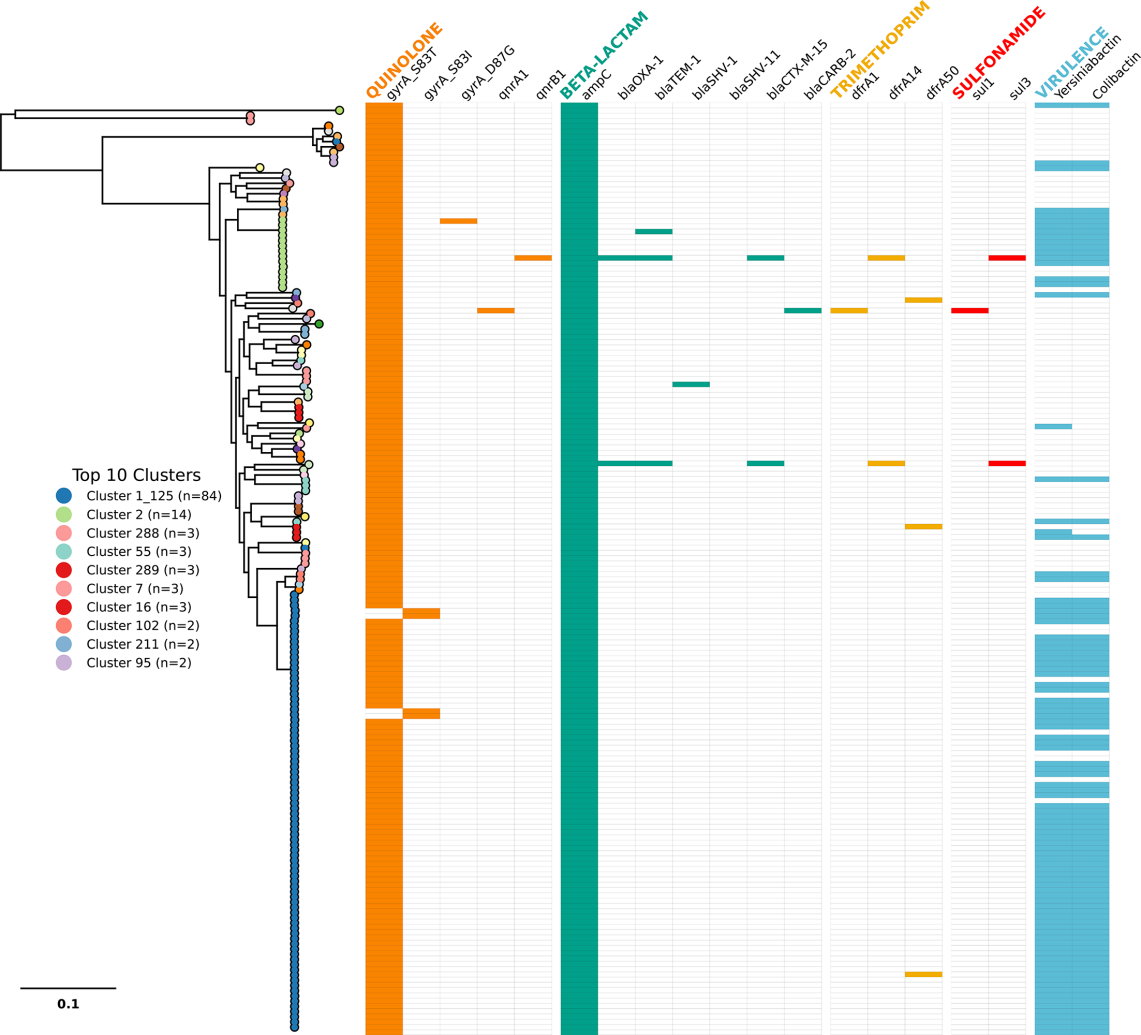
Maximum likelihood phylogenetic tree of New Mexico *K. aerogenes* isolates showing presence or absence of AMR and virulence genes. The isolates (*n*=177) are colour-coded based on PopPunk cluster, and the presence or absence is coloured based on drug class or virulence factor.

To investigate the genetic relatedness and population structure of our isolates sequencing on 177 isolates, we contextualized our New Mexico isolates within a representative set of 762 global genomes [23]. The broad distribution of the New Mexico isolates across the phylogeny indicates that the *K. aerogenes* population within Albuquerque is diverse and reflective of the global diversity of the species (Fig. 2). Additionally, we typed the resulting high-quality *K. aerogenes* genomes using our PopPunk database. These isolates were diverse, spanning 61 distinct PopPUNK clusters (PP) (Fig. 2); however, the majority of isolates belonged to two distinct clusters, corresponding to PopPunk cluster PP-1_125 (ST93, *n*=84) and PP-2 (ST4, *n*=14) (Table 1, Fig. 3). This is notable, as ST4 and ST93 are consistently identified as the dominant global and pandemic clones of *K. aerogenes*, broadly distributed worldwide and strongly associated with infections and outbreaks [23]. These lineages are characterized by their high prevalence of key virulence factors, such as yersiniabactin and colibactin (often carried on the ICEKp10 element), and their frequent association with antimicrobial resistance [7,23]. We also examined pairwise SNP values, which ranged from 3 to 155,046 SNPs (median=21,064 SNPs), which reinforces the breadth of diversity sampled across our isolates.

**Table 1.** Five most prevalent clusters identified in New Mexico and corresponding MLST, cgMLST clonal cluster classifications and the number of isolates in each

PopPunk cluster	Associated MLSTs	cgMLST cluster	No. of NM isolates
**1_125**	388, 57, 571	CC1, CC3	84
**2**	594, 4	CC2	14
**7**	92	CC6	4
**16**	519, 533	CC29	3
**55**	128	CC18	3

**Fig. 4.**
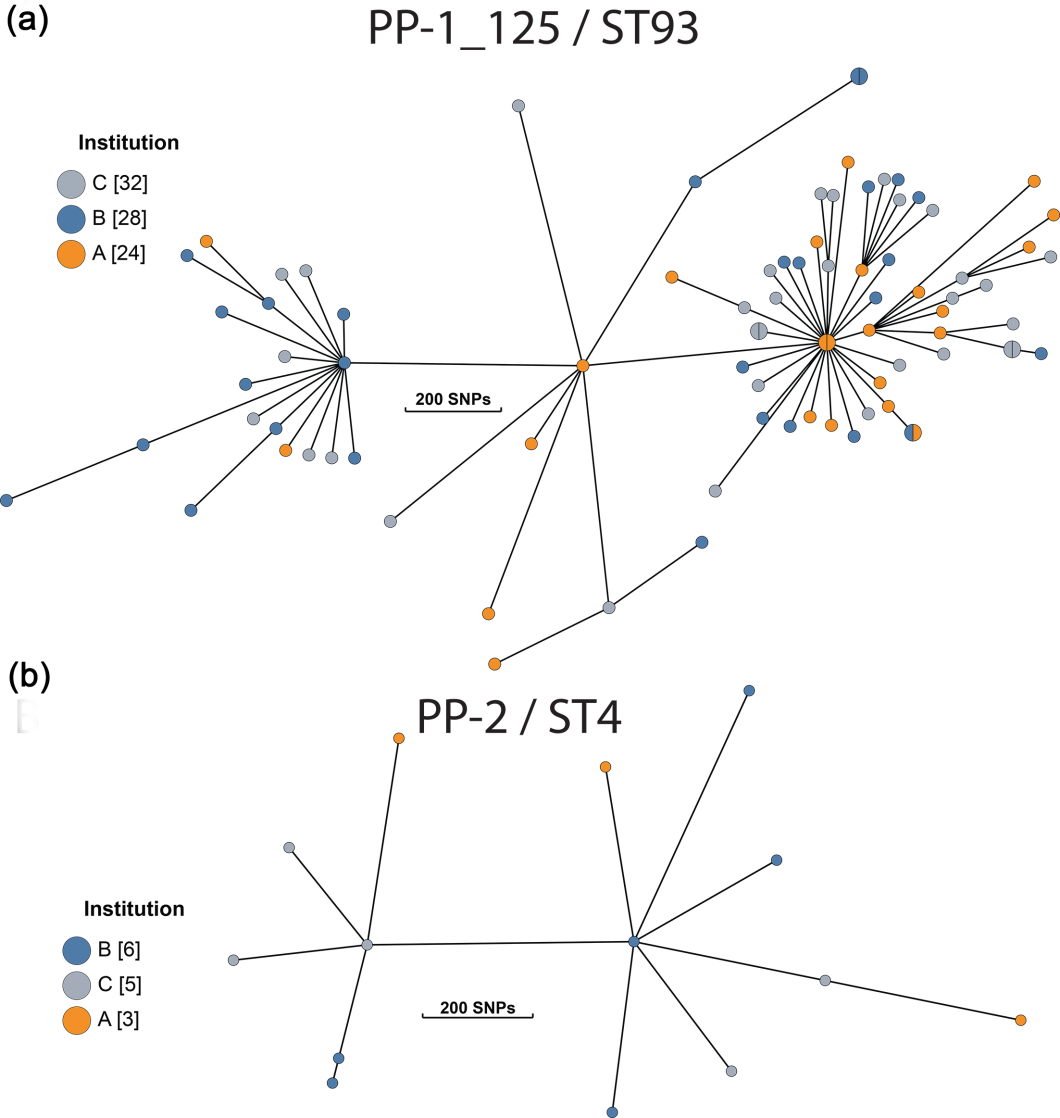
Minimum spanning tree for (a) PP-1_125 and (b) PP-2 lineages. The nodes are collapsed at 30 SNPs, and the size of the circles is proportional to the number of strains within a given node. Nodes are coloured by institution. The number of samples per institution is indicated in brackets. Branch lengths correspond to the number of SNPs.

As over half of our isolates (98/177) were classified as either PP-1_125 or PP-2, this provided an opportunity to characterize within-cluster diversity metrics. The genetic variation within these clusters was significant. We find pairwise SNP differences in PP-1_125 (*n*=84) range from a minimum of 3 SNPs to a maximum of 774 SNPs (median=442 SNPs). For PP-2 (*n*=14), we find SNPs range from 27 to 569 SNPs (median=410 SNPs). We visualized these relationships using minimum-spanning trees (Fig. 4).

### Local transmission chains

We defined putative transmission clusters using a threshold of ≤30 pairwise SNPs. While stricter thresholds (e.g. <21 SNPs) are often used to infer direct transmission in related species like *Klebsiella pneumoniae* [24], we employed a slightly higher cutoff to capture broader transmission networks and recent shared ancestry. Surprisingly, we found only four pairs of individuals that meet our criteria (Fig. S3). These four SNP distances were 3, 6, 19 and 27 SNPs. Three of the four involved pairs were between different institutions. These transmission pairs ranged from 14 to 260 days apart, with the smallest 3 SNP difference found in the pair that was 260 days apart. Though they were rare, our data do provide evidence of possible transmission of *K. aerogenes* both within and between healthcare facilities within the Albuquerque metro area.

### Serial sampled individuals

**Fig. 5.**
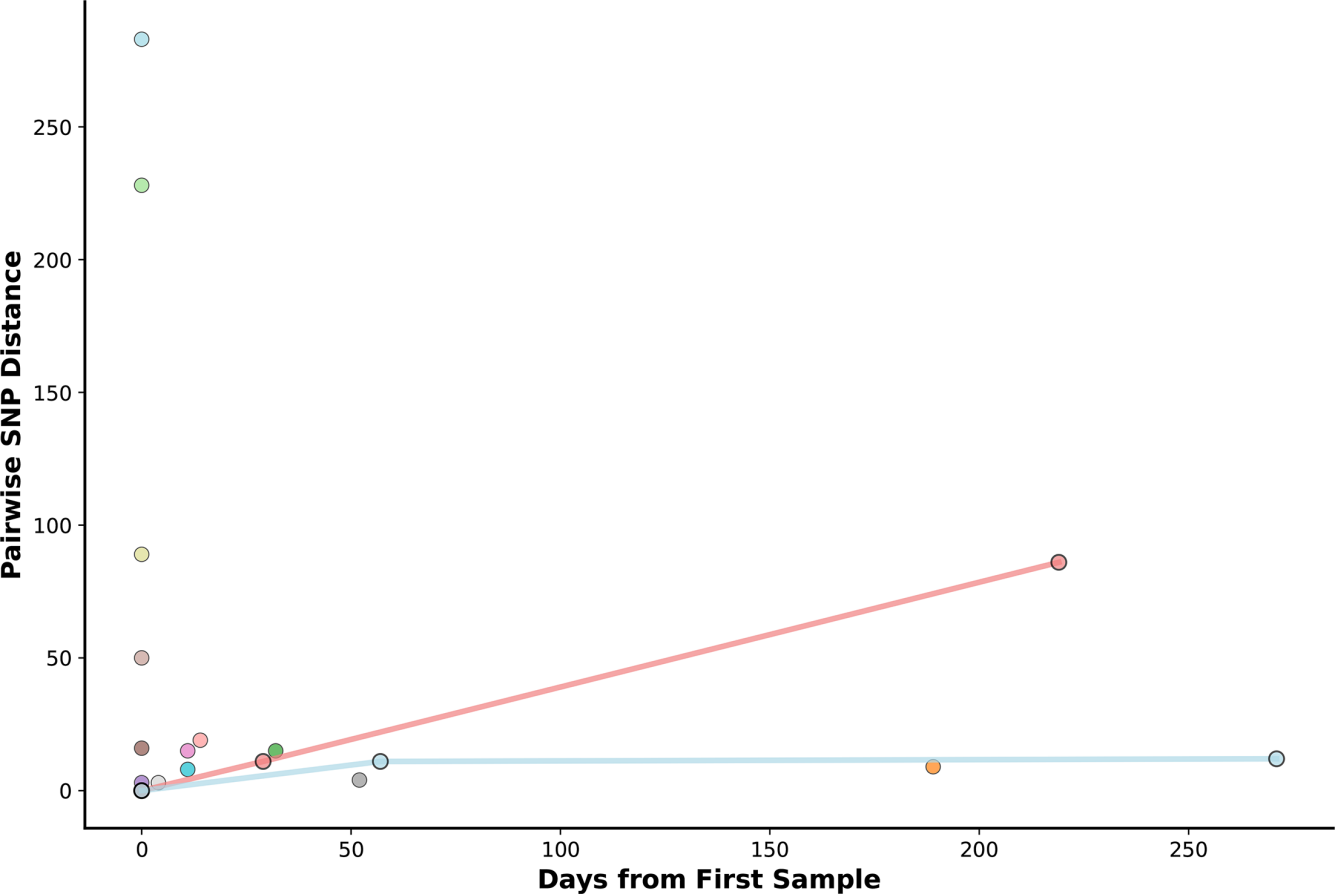
Pairwise SNP distances from serially sampled individuals. The number of pairwise SNPs is on the *Y*-axis, and the days between samples are shown on the *X*-axis. Two patients had three samples each, represented by the lines connecting these points.

We examined within-host diversity using multiple isolates from 15 individuals (Fig. 5). While all serial isolates from the same individual shared identical PP and ST profiles, the pairwise SNP diversity ranged from 3 to 283 (median=15). The time between sample collections varied from the same day to 271 days, with most pairs (*n*=12/19) collected within 50 days. Unexpectedly, we found greater SNP diversity in samples collected less than a week apart (median=50 SNPs, range=3–283) compared to those collected over a week apart (median=12 SNPs, range=4–86).

Our analysis of serially sampled isolates with elevated pairwise SNP distances identified a single case consistent with a putative mismatch-repair-associated hypermutator phenotype. Among five serial comparisons with >50 SNP differences, only one individual (Person 62) harboured non-synonymous mutations in *mutS* located within the conserved ATPase domain of the protein, associated with hypermutator phenotypes [25,26]. Both isolates from this individual carried two missense substitutions (Thr544Ile and Ile573Leu) and exhibited an 89-SNP difference despite being sampled on the same day. In contrast, the remaining high-SNP serial comparisons lacked disruptive *mutS* mutations or contained only late C-terminal or connector-domain variants, which are not typically associated with strong mutator phenotypes [25,26]. These findings suggest that elevated SNP distances observed in most serial comparisons are unlikely to be driven by mismatch-repair deficiency, whereas the Person 62 isolates represent a plausible case of *mutS*-associated hypermutation.

Two distinct evolutionary patterns emerged from the data. A subset of individuals showed consistently low SNP diversity (~10–15 SNPs) across all timepoints, suggesting clonal infections with limited mutation accumulation over time. However, other individuals maintained high SNP diversity (>80 SNPs) even at extended timepoints beyond 150 days. This suggests that initial infections may often be polyclonal. Over time, this complexity resolves in one of two ways: either competitive exclusion leads to a single, persistent lineage that evolves clonally, or multiple strains establish a stable, long-term coexistence within the host, potentially occupying distinct anatomical niches or surviving under different selective pressures such as varying antibiotic exposures.

### Antimicrobial resistance and virulence genes

*K. aerogenes* is unique among *Klebsiella* species for possessing an intrinsic, chromosomally encoded class C beta-lactamase (*ampC*). This inducible *ampC* confers broad cephalosporin resistance [7,27] (Fig. 3). As expected, we detected this gene across all our New Mexico (NM) isolates. Beyond this intrinsic resistance mechanism, we identified additional beta-lactamase genes in only five isolates. The isolates harboured narrow-spectrum beta-lactamases such as blaOXA-1 (*n*=2), blaCARB-2 (*n*=1), blaTEM-1 (*n*=3) and blaSHV-1 (*n*=1). Importantly, we detected two isolates that harboured the extended-spectrum beta-lactamse (ESBL) gene blaCTX-M-15. These two ESBL-positive isolates carried multiple beta-lactamase genes (blaOXA-1, blaTEM-1, blaCTX-M-15). We found that the blaCTX-M-15 and bla-TEM-1 were co-localized on an IncF-type plasmid that had 100% identity and 100% query coverage matches to plasmids found in other *K. aerogenes* (CP103640.1) and *K. pneumoniae* (OZ111394.1) isolates. Importantly, we did not detect any isolates with carbapenemase-encoding genes in our NM dataset.

Fluoroquinolones are one of the most commonly used antibiotics for outpatient care, particularly for uncomplicated UTIs [28]. All NM isolates harbour mutations within *gyrA* at position Ser-83, which is part of the quinolone resistance determining region [29]. Mutations at gyrA_S83 have been described previously as conferring reduced susceptibility to fluoroquinolones [30,31]. Nearly all isolates have a gyrA_S83T (*n*=175), while the remaining four isolates harbour gyrA_S83I. Although the *K. aerogenes* type strain (KCTC 2190) encodes serine at position 83, our analysis of global genomes indicates that threonine is present in 94% (*n*=715/762) of the population, suggesting threonine at this position may be the wild-type allele. However, these two mutations in *gyrA* specifically have been shown to confer reduced susceptibility to quinolones [29]. Two isolates have also acquired *qnr* genes – either *qnrA1* or *qnrB1*. Both of these isolates also happen to carry *dfrA* genes and have at least one ESBL gene. Trimethoprim resistance genes (*dfrA1* and *dfrA14*) were detected in only six isolates. Other resistance mechanisms were less common, with scattered tetracycline resistance (*tetA* genes) and sulphonamide resistance (*sul1*, *sul2*) genes present in a subset of isolates.

A variety of virulence factors have been identified within *K. aerogenes*, with the most important being the metallophore system yersiniabactin (*ybt*) and the genotoxin system colibactin (*clb*) [7]. These genes typically co-locate, and indeed, we detected yersiniabactin in 98 and colibactin in 96 isolates out of 171 total isolates (Fig. 3). Of these 98 *ybt*-positive isolates, 74 belonged to the PP-1_125, and an additional 12 belonged to PP cluster 2. Colibactin and yersiniabactin presence was almost exclusive to these two clusters, suggesting these factors may be involved in the success of these pandemic *K. aerogenes* lineages. Notably, the alleles for both virulence factors were homologous to those characteristic of *K. pneumoniae*. Outside of ST4 and ST93 clusters, only six isolates across six STs carried both *ybt* and *clb* – specifically ST15, ST116, ST151, ST503, ST525 and ST535.

### Putative adaptive resistance mutations

**Fig. 6.**
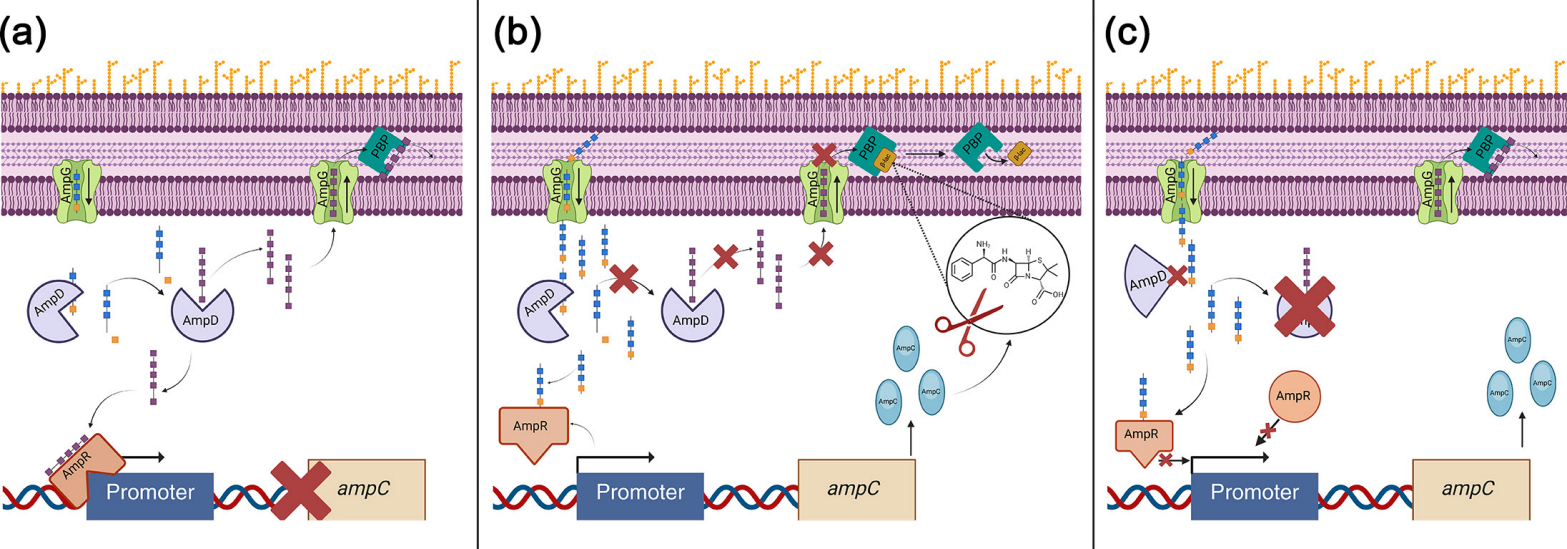
(a) Schematic of a functional AmpC regulatory pathway in the absence of beta-lactams. (b) A functional AmpC regulatory pathway in the presence of beta-lactams and (c) potential dysfunctional upstream elements in a mutated AmpC pathway.

The AmpC pathway facilitates peptidoglycan recycling due to natural breakdown and can be induced in the presence of beta-lactam antibiotics [32] (Fig. 6). Disruptions to the upstream regulatory elements of AmpC can lead to constitutive and even hyperexpression of AmpC beta-lactamase [33]. Specific mutations within AmpD [34] and AmpR [35] can cause high-level, constitutive AmpC expression [34,35], thereby contributing significantly to broad beta-lactam resistance phenotypes, including decreased susceptibility to carbapenems [5,36] (Fig. 6).

**Fig. 7.**
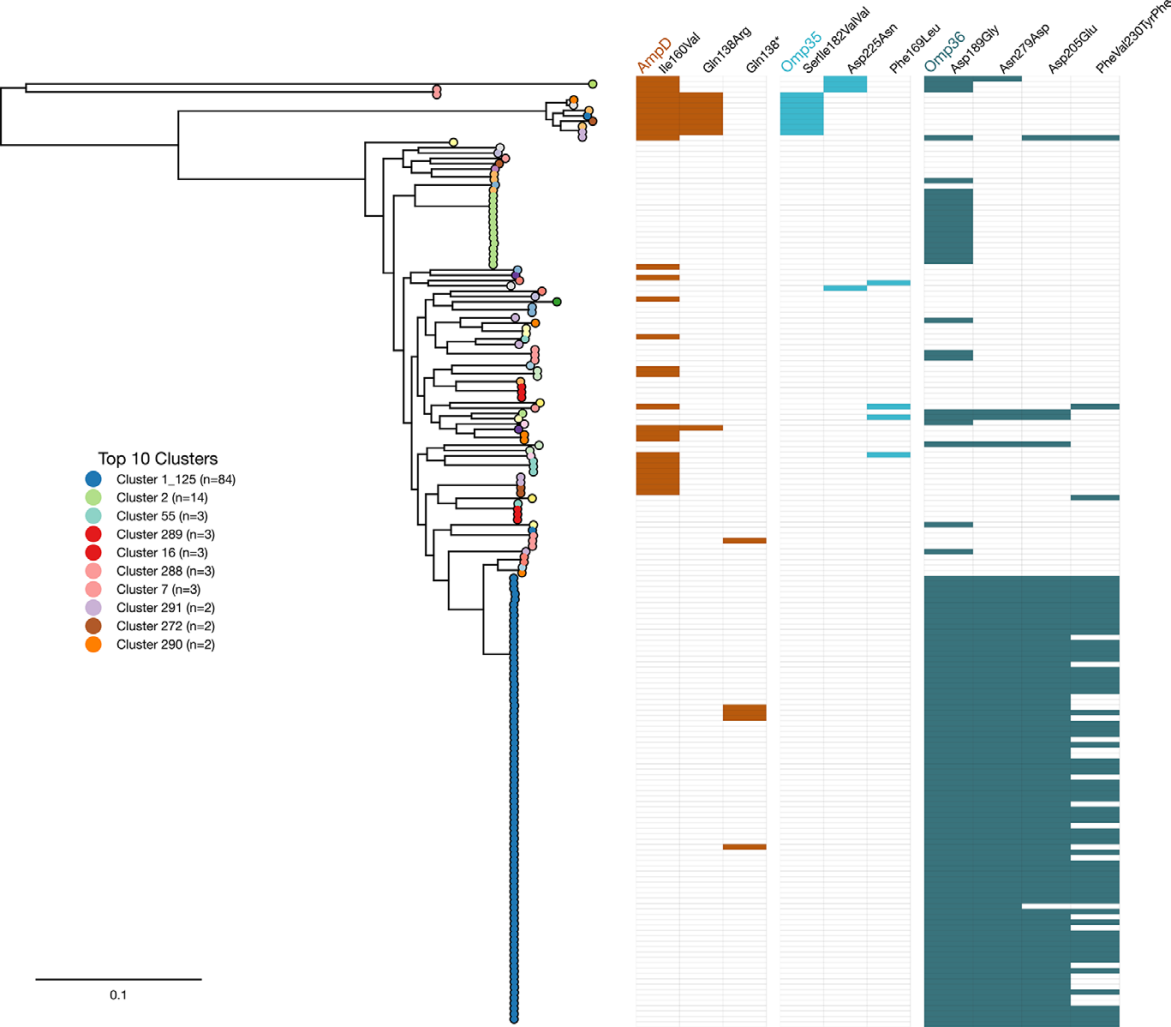
Maximum likelihood phylogenetic tree of New Mexico *K. aerogenes* isolates showing presence or absence of mutations in AmpD, Omp36 and Omp36. The isolates (*n*=177) are colour-coded based on PopPunk clusters. Amino acid changes and the position within the protein are shown.

In our NM isolates, we identified 29 unique non-synonymous mutations in *ampD*. The most prevalent mutation was Ile160Val (*n*=30), followed by Gln138Arg (*n*=5) and a Gln138X nonsense mutation (*n*=5) (Fig. 7). This pattern mirrored that of the larger *K. aerogenes* dataset, where Ile160Val was the most prevalent (*n*=310), followed by Gln138Arg (*n*=113), with the Gln138X nonsense mutation ranking fifth (*n*=30) (Fig. S4). Of the 55 AmpD truncations identified across the entire *K. aerogenes* dataset, 30 occurred at Gln138 (54.5%). The Gln138X nonsense mutation results in protein truncation and has been associated with a CR phenotype in a clinical isolate [9]. Crucially, we captured the *de novo* acquisition of this mutation in Person 37, where the patient’s initial isolate carried a wild-type *ampD*, and the subsequent isolate collected 11 days later (separated by 15 SNPs) had acquired the Gln138* nonsense mutation. While Gln138Arg was common in both NM and global datasets, its phenotypic impact remains unknown [7]. We identified three additional singleton nonsense mutations in our NM isolates at positions Cys108X, Tyr125X and Gln123X. The most frequent mutation, Ile160Val, was previously described in one CR isolate [9], but sparse data exist regarding its functional impact.

Outer membrane porins are critical for bacterial permeability and antibiotic uptake, and mutations in these genes represent a key mechanism of adaptive resistance in *K. aerogenes*. We identified non-synonymous mutations in Omp35 (also known as OmpK35) in 19 NM strains, consisting of 31 unique mutations. We identified SerIle182ValVal (*n*=8), Phe169Leu (*n*=4) and Asp225Asn (*n*=4) as the three most prevalent mutations in our NM isolates. Two PP-114 (ST507) strains and a single PP-8 (ST103) strain contained eight mutations within this gene. We did not identify any truncations in the outer membrane porin Omp35 in our NM isolates.

Omp36 is an ortholog of OmpC and is critical for outer membrane permeability and is a frequent site for adaptive resistance mutations in *K. aerogenes* [9]. We detected non-synonymous mutations in *omp36* in 162 of 177 (92%) strains within our NM isolates, corroborating the high prevalence of mutations within this gene [7]. We identified highly prevalent mutations such as Asp189Gly (*n*=112), Asn279Asp (*n*=88), Asp205Glu (*n*=87) and PheVal230TyrPhe (*n*=64) (Fig. 7). The first three mutations were found in all PP-1_125 strains (*n*=84), with the exception of a single strain lacking the Asp205Glu mutation. All PP-2 strains (*n*=14) all harboured Asp189Gly coupled with Ile59Val. We identified only two truncation events in *omp36*, one strain harbouring Gln66X and another with Gln294X, both of which were in PP-1_125.

## Discussion

This study provides the first comprehensive genomic epidemiological analysis of clinical *K. aerogenes* isolates in New Mexico. Our findings reveal a diverse local population that is dominated by two globally recognized pandemic lineages, PP-1_125 (ST93) and PP-2 (ST4). This mirrors the global landscape, suggesting that the success of these high-risk clones is not confined to major international hubs but extends to regional healthcare systems. The predominance of these lineages is concerning, as they are strongly associated with key virulence factors, yersiniabactin and colibactin, which were highly prevalent in our dataset and found predominantly within these two clusters. Genomic evidence for recent, direct nosocomial transmission was surprisingly scarce, with only four putative transmission events identified. This suggests that while high-risk clones are pervasive, the Albuquerque *K. aerogenes* burden may be driven more by sporadic, community-acquired cases or independent acquisitions within healthcare settings rather than large, sustained nosocomial outbreaks. It is plausible that our sampling missed intermediate links in transmission chains. Increased sampling across the healthcare network may reveal more direct transmission links than we described here from our retrospective convenience sampling.

Further complexity is revealed by the significant genetic diversity observed within individual patients over time. The finding of higher genetic diversity in samples collected in close temporal proximity strongly suggests that a significant fraction of patients are initially infected with multiple, distinct strains. The identification of a putative *mutS*-associated hypermutator phenotype in one individual highlights the potential for mismatch-repair defects to drive rapid strain diversification. Our analysis uncovered two distinct patterns of within-host evolution: stable, clonal persistence with minimal change, and long-term maintenance of polyclonal infections with high genetic diversity (>80 SNPs). This polyclonality may have clinical implications, as a diverse infecting population may harbour varied resistance and virulence profiles, potentially complicating antibiotic therapy and enabling the selection of more resistant or virulent subpopulations. The subsequent resolution to either a single clonal lineage or a stable, long-term coexistence of strains underscores the dynamic interplay among the host immune system, antibiotic pressures and microbial competition.

To facilitate robust genomic typing, we developed a novel PopPUNK database for *K. aerogenes*, which demonstrated high concordance with existing MLST and cgMLST schemes while offering greater flexibility. Our PP clusters demonstrated both liberal and conservative behaviour relative to cgMLST assignments. In 3 cases (10.1% of isolates), PopPUNK merged closely related groups sharing whole-genome similarity, while in 45 cases (25.0% of cgMLST groups), it split diverse cgMLST complexes into finer subdivisions. These patterns were supported by our phylogenetic analyses (Fig. S2). This dual behaviour reflects PopPUNK’s consideration of both core and accessory genome variation. Strains with nearly identical whole-genome profiles may be united despite minor allelic differences in cgMLST loci. Conversely, strains sharing cgMLST designations may be separated when they have divergent accessory genomes or evidence of recombination. The high concordance between methods (99.0% of PP clusters corresponding to single cgMLST groups, representing 89.9% of isolates) validates the biological meaningfulness of both approaches. The flexibility of PopPUNK clustering provides a practical framework for rapid lineage assignment of new *K. aerogenes* isolates.

This study has several limitations. As a retrospective convenience sample, it may not fully represent the true prevalence and diversity of *K. aerogenes* in the region. Furthermore, the lack of linked clinical outcome data and phenotypic susceptibility testing prevents direct correlation of genotype with clinical phenotype and patient outcomes. Despite these limitations, our work establishes a critical genomic baseline for *K. aerogenes* in New Mexico. These findings underscore the need for expanded surveillance across New Mexico to better understand *K. aerogenes* transmission dynamics, resistance evolution and clinical impact.

## Supplementary material

10.1099/mgen.0.001650 Uncited Supplementary Material 1.

10.1099/mgen.0.001650 Uncited Table S1.

10.1099/mgen.0.001650 Uncited Table S2.

## Abbreviations

CR: carbapenem-resistant
CR-KA: carbapenem-resistant *Klebsiella aerogenes*
DBSCAN: Density-Based Spatial Clustering of Applications with Noise
STs: sequence types
UTIs: urinary tract infections
