# Supplementary material for: Genomic analysis of Klebsiella aerogenes circulating in New Mexico
Source: Microb Genom. 2026 Feb 24;12(2):001650. doi: 10.1099/mgen.0.001650 (PMC12958145; doi:10.1099/mgen.0.001650)
Supplement: Uncited Supplementary Material 1. [file mgen-12-01650-s001.pdf]

Supplementary Figures

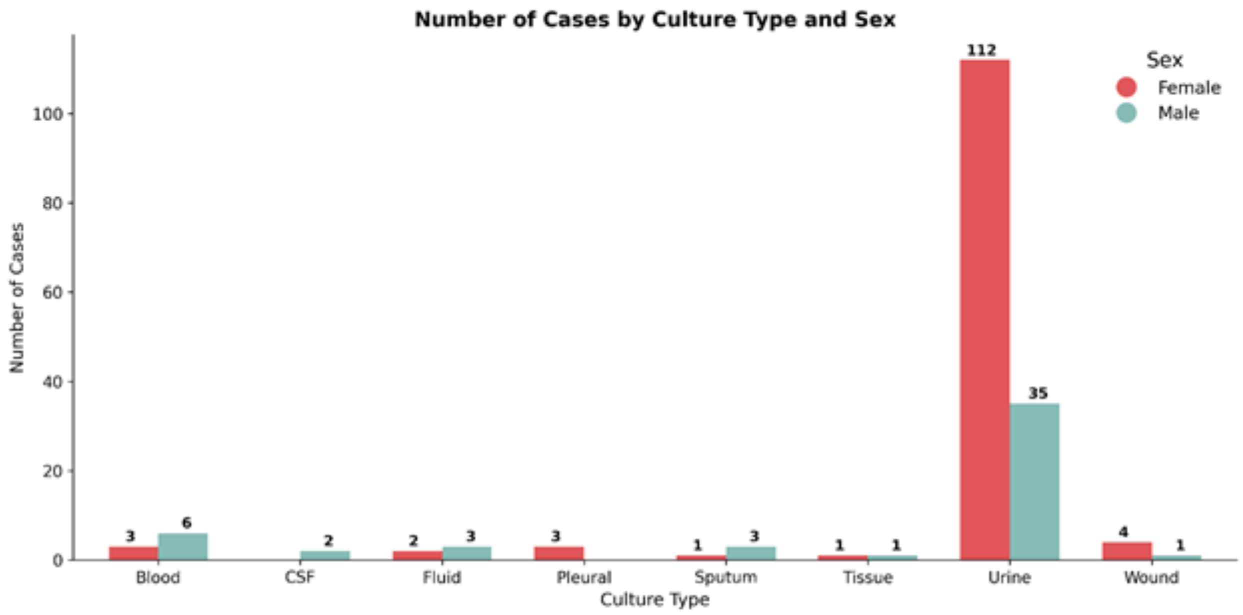

Supplementary Figure 1. Number of cases broken down by sample type and stratified by sex.

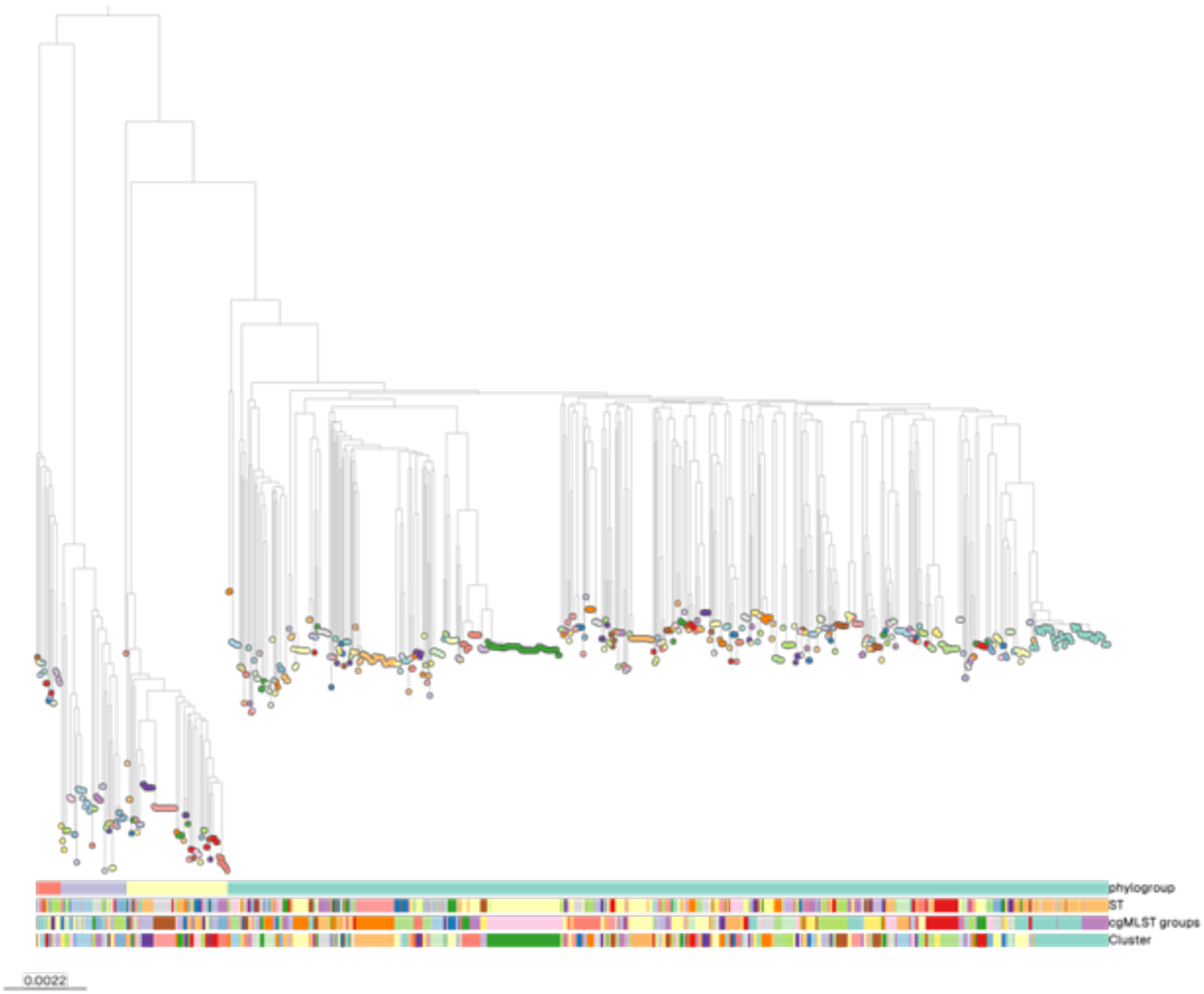

Supplementary Figure 2. Phylogenetic tree of 763 *Klebsiella aerogenes* used to create the PopPunk database. Nodes are colored by PopPunk (PP) cluster. The phylogroup, ST, cgMLST, and PP clusters are indicated by color bars.

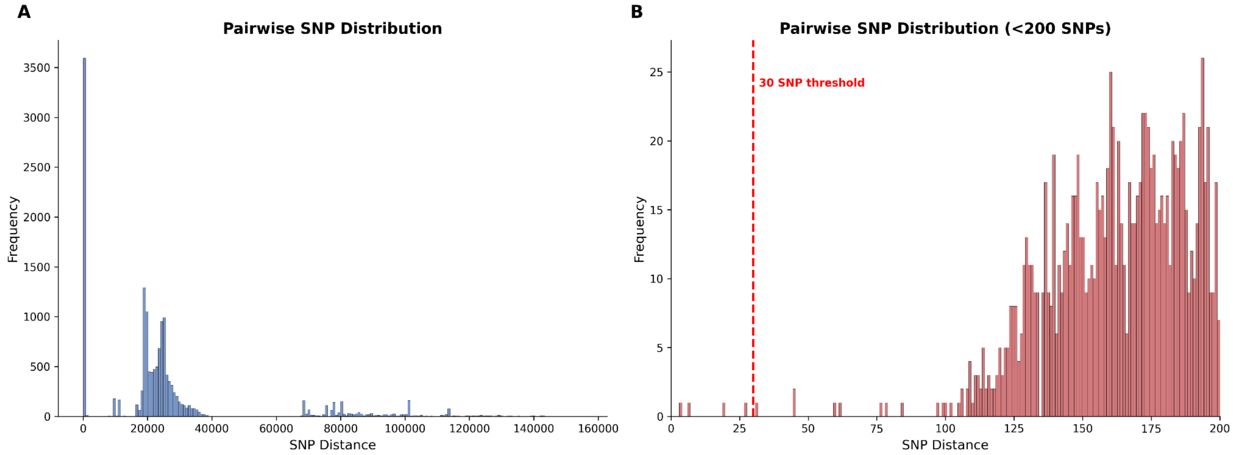

Supplementary Figure 3. Distribution of pairwise SNP differences between *Klebsiella aerogenes* isolates from different patients. A. Full distribution of pairwise single nucleotide polymorphism (SNP) differences for all unique inter-person isolate pairs. **B.** Zoomed-in view of the distribution focusing on pairs with fewer than 200 SNPs. The red dashed line denotes a 30-SNP threshold.

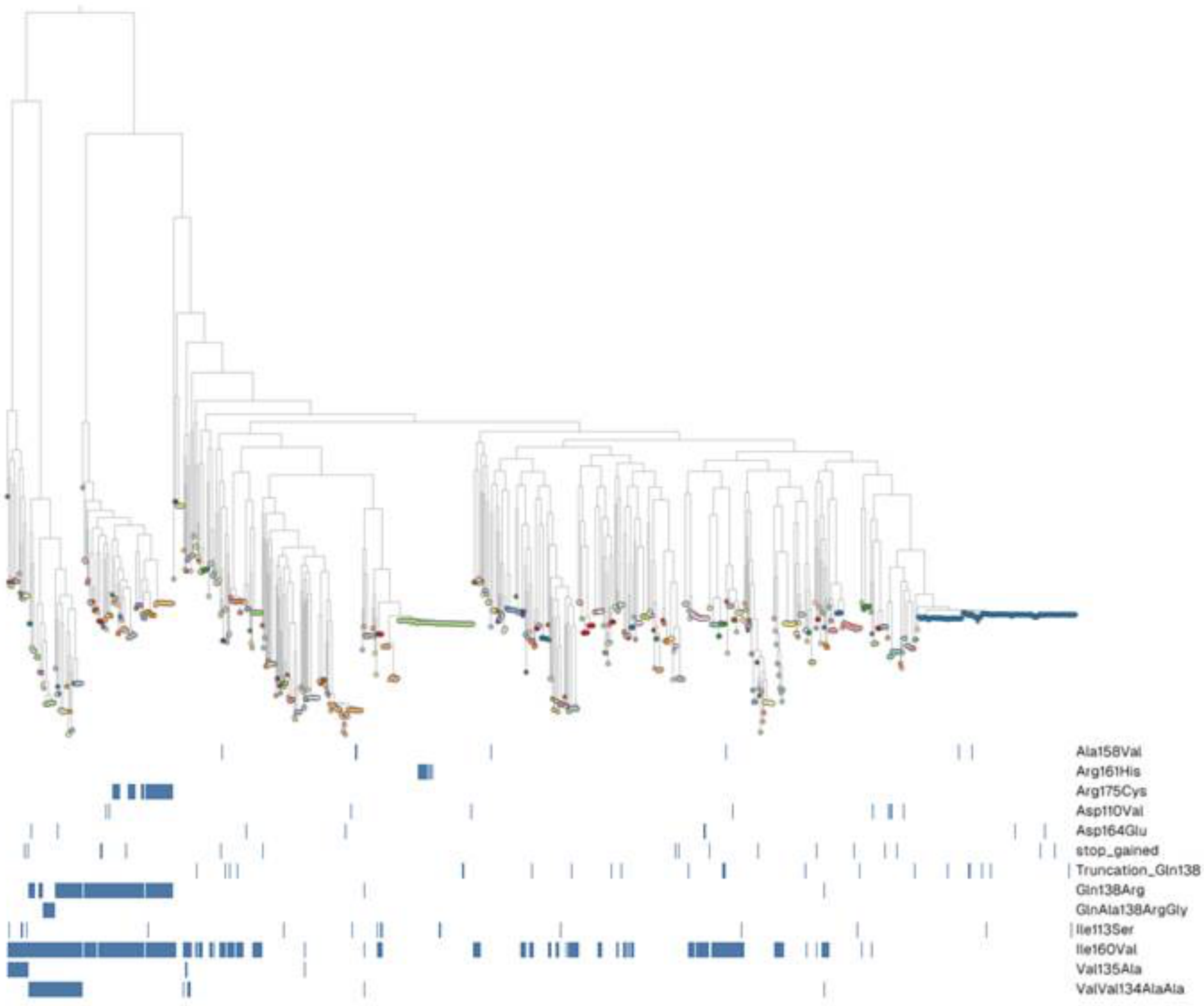

Supplemental Figure 4. Global comparison of AmpD mutations. A maximum likelihood phylogenetic tree of *Klebsiella aerogenes* isolates from PopPunk database and New Mexico (n=948) with corresponding presence/absence of the most prevalent mutations in AmpD.
